# The Effect of High-Voltage Power Lines on Magnetic Orientation of Domestic Dogs

**DOI:** 10.3390/ani15243534

**Published:** 2025-12-08

**Authors:** Nataliia S. Iakovenko, Kateřina Benediktová, Jana Adámková, Vlastimil Hart, Hana Brinkeová, Miloš Ježek, Tomáš Kušta, Vladimír Hanzal, Petra Nováková, Hynek Burda

**Affiliations:** 1 Faculty of Forestry and Wood Sciences, Czech University of Life Sciences Prague, Kamýcká 129, 165 21 Prague, Czech Republic; benediktovak@fld.czu.cz (K.B.); adamkovaj@fld.czu.cz (J.A.); hart@fld.czu.cz (V.H.); brinkeova@fld.czu.cz (H.B.); jezekm@fld.czu.cz (M.J.); kusta@fld.czu.cz (T.K.); burda@fld.czu.cz (H.B.); 2 Department of Biology and Ecology, University of Ostrava, Chittusiho 10, 701 03 Ostrava, Czech Republic

**Keywords:** alternating magnetic field, geomagnetic storms, magnetoreception, mammal ethology, spontaneous magnetic alignment

## Abstract

Domestic dogs can sense the geomagnetic field (GMF), spontaneously aligning their bodies along its axis, altering the alignment’s pattern during geomagnetic disturbances. Whether anthropogenic magnetic fields (MF) from high-voltage power lines (PL) influence this behavior remains unclear. We investigated the effects of alternating MF generated by PL on spontaneous magnetic alignment in 36 dogs. Behavior was recorded under north–south (NS) and east–west (EW) oriented PL and compared with control conditions lacking anthropogenic MF. Each dog’s mean alignment angle relative to magnetic north was calculated from >50 measurements per condition, and Grand Means (GMs) were derived. Under control geomagnetically calm conditions, alignment was bimodal (GM = 23°/203°), while geomagnetic storms caused significant shifts and increased angular dispersion. Under NS-oriented PL, alignment remained bimodal (GM = 5°/185°), but under EW-oriented PL it became trimodal (Likelihood ratio test for multimodality: nodes = 3, *p* = 0.042; GM = 103°/283°). These differences were statistically significant (LME for linearized angles: *p* < 0.001 for control vs. NS PL and control vs. EW PL). Our results demonstrate that dogs maintain directional alignment under PL exposure, with orientation patterns corresponding to the direction of both MF and PL, which suggests a potentially complex impact involving non-magnetic cues.

## Simple Summary

Domestic dogs have been reported to sense the Earth’s magnetic field, aligning their bodies along its axis and altering this alignment during geomagnetic disturbances. Building on previous findings in cattle and deer, we tested whether high-voltage power lines disrupt directional alignment in dogs, presumably by interfering with a magnetoreceptive system tuned to the geomagnetic field. Contrary to our expectations, dogs still exhibited axial alignment under the power lines. However, both the direction and pattern of their alignment shifted markedly directly under the power line wires. Our results suggest that, rather than simple disruption, power lines may induce a complex interplay of magnetic and non-magnetic cues modulating dogs’ orientation behavior.

## 1. Introduction

Considerable progress has been made during recent decades in understanding how humans and animals detect and respond to magnetic fields (MFs) of various strengths and dynamics, including alternating MF below 300 Hz (classified as extremely low frequency) generated by high-voltage power lines (PL). The effects of MF on human health have been investigated primarily in relation to carcinogenic [1,2,3,4], cardiovascular [5], reproductive and developmental outcomes [6,7,8,9,10,11]. Behavioral and physiological changes in animals exposed to alternating MF of varying intensities have also been reported [12,13,14]. In contrast, the influence of alternating MF on spatial orientation in animals—particularly in relation to their natural magnetic sense or *magnetoreception*—remains poorly understood and has received limited attention [15,16,17,18,19,20].

Magnetoreception is most widely studied in the context of perceiving the geomagnetic field (GMF) with magnetic induction varying across Earth from around 20 to 70 µT. Many vertebrates rely on GMF for navigation during homing and migration [20,21,22,23,24]. In addition to migratory birds, more than 30 mammal species have been shown to use magnetic clues for long-distance navigation, hunting, and other orientation-related behaviors [25,26,27,28,29]. Recently, magnetoreception has been demonstrated in domestic dogs, which are readily available and suitable test subjects for behavioral experiments [30,31,32,33].

Several mechanisms have been proposed to explain magnetoreception in animals, with the most frequently addressed being the radical-pair mechanism and magnetite-based receptors. The radical-pair mechanism studied primarily in migratory songbirds, insects, and more recently in mammals [34,35,36] relies on photochemical reactions in blue-light–sensitive flavoproteins (cryptochromes) located in the retinal tissue. These reactions lead to the formation of radical pairs in flavin adenine dinucleotide and tryptophan ([FAD•− TrpH•+]), whose interconversion between singlet and triplet states can be influenced by weak magnetic fields, thereby generating directional responses in retinal neurons. This mechanism is light-dependent, provides information on the inclination rather than the polarity of the geomagnetic field (GMF), and therefore, cannot distinguish poleward direction. In contrast, magnetoreception based on the alignment of magnetite crystals, Fe_3_O_4,_ does not require light and can detect the polarity of the horizontal GMF component. The evidence for this mechanism has been reported in bats, Ansell’s mole-rats, birds, and migratory fish [37,38,39].

One external manifestation of magnetic sensitivity is the spontaneous aligning of the body axis along GMF lines, commonly termed *‘magnetic alignment’* [18]. This phenomenon was first documented in insects and later in fish, caudate amphibians, reptiles, birds, and mammals [20,30,40,41,42,43,44,45]. It was hypothesized that the magnetic alignment aids in reconciling external environmental cues with the animal’s internal cognitive map, thereby reducing its spatial complexity and demands on memory [46,47]. In dogs, excretion serves as a form of scent-marking, functioning as a ‘bulletin board’ or even ‘property line’ [48]. Alignment during urination or defecation may thus assist in marking a location within the animal’s cognitive map and in facilitating its later recall.

Natural phenomena from beyond Earth (solar wind and flares) cause frequent fluctuations in the GMF known as geomagnetic storms. Exposure to GMF during such periods of disturbances has been shown to affect spontaneous magnetic alignment in animals; in dogs, rapid changes in GMF declination during GMF disturbances shifted alignment direction, increasing scattering of bearings to the state of almost disrupted alignment during strong magnetic storms [30]. Anthropogenic alternating MF generated by high-voltage PL superimposes on static GMF, producing magnetic disturbances that can disrupt spontaneous alignment of ruminants [49], with bearings scattering effect attributed not only to oscillations in the MF direction but also to ~12% oscillations in MF intensity—an effect inconsistent with both magnetite-based polarity compass and light-dependent inclination-based magnetoreception.

**Figure 1:**
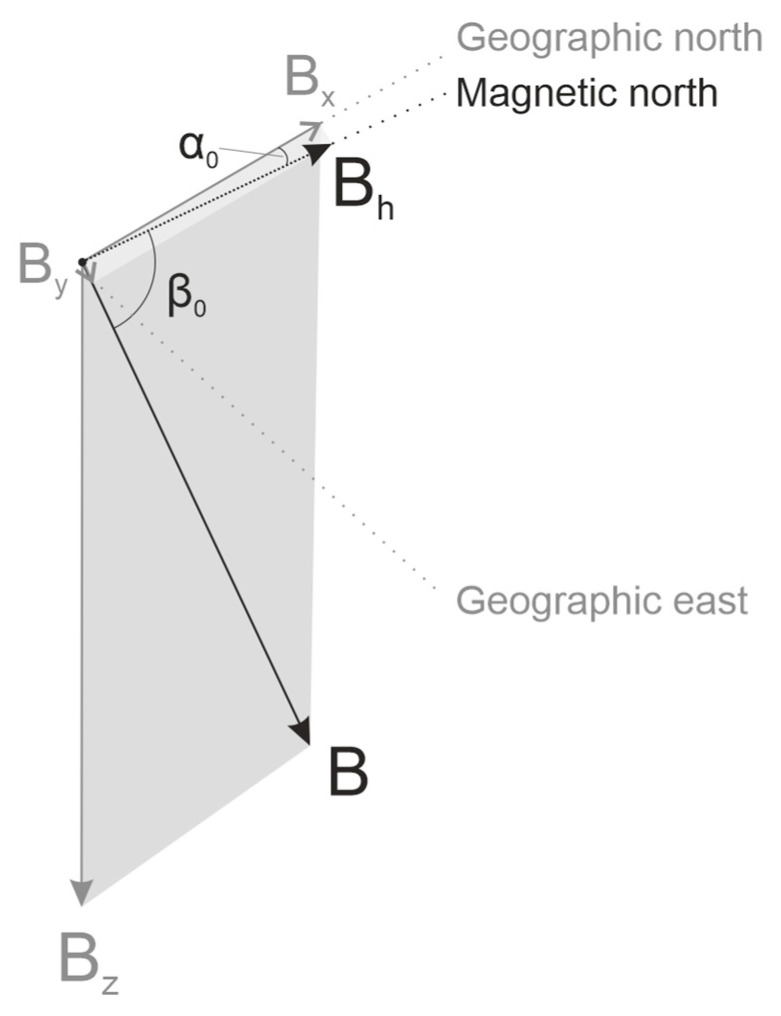
Schematic representation of the geomagnetic field (GMF) in the Northern hemisphere, Central Czechia. The relative magnitudes of the vectors are maintained according to their magnetic induction values. The total vector of the Earth’s MF magnetic induction (**B**) is a sum of two horizontal components (**B**_h_ = **B**_x_ + **B**_y_), and a vertical component **B**_z_. The angle α_0_ between the axis pointing to the true (geographic) north, and **B**_h_, corresponding to the direction of the compass needle pointing to magnetic north, is the magnetic declination of GMF, often referred as *D*. In the northern hemisphere **B**_z_ is pointing downwards perpendicularly to **B**_h_, thus GMF lines point down with the angle β_0_ indicating its magnetic inclination, designated as *I*. The length of the vector components is proportional to their magnetic induction measured in µT.

**Figure 2:**
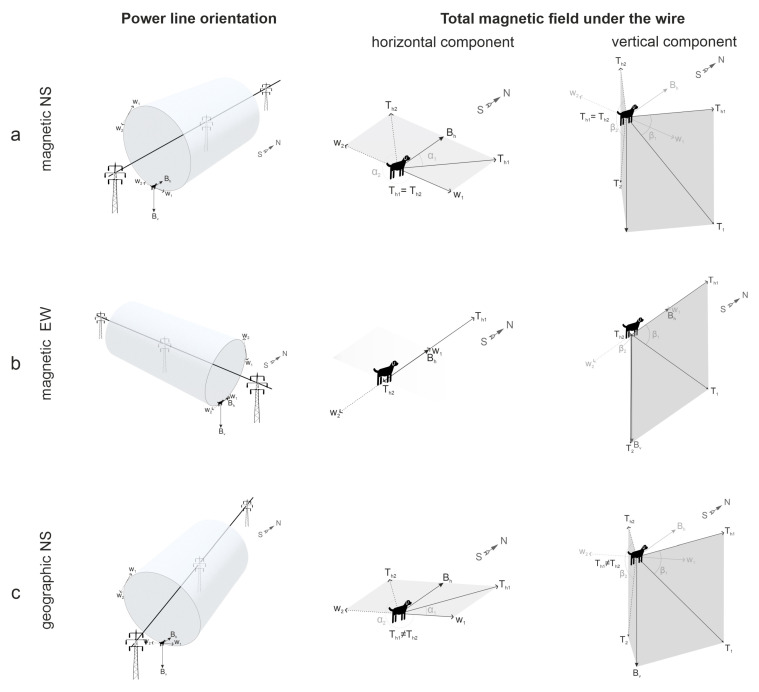
Magnetic field characteristics under high-voltage power lines (PL). The length of the vector components is proportional to their magnetic induction. The direction towards magnetic north indicated by the north–south arrow corresponds to that of **B**. The vector of magnetic induction of the wire **W** right under PL has only a horizontal component oscillating between **W**_1_ and **W**_2_ (and **W**_1_ = **W**_2_). Lines of the wire’s MF encircling it in an annular manner are depicted as cylindrical shapes, **W** is pointing tangentially to them. The total vector **T** = **B** + **W** periodically changes direction, oscillating between **T**_1_ and **T**_2_ (alternating current frequency is 50 Hz in Central Czechia). (**a**) An idealized example where a dog stands right under the wire that goes strictly parallel to the GMF direction (i.e., to the magnetic, not geographic, NS axis). **W** is perpendicular to the horizontal vector **B**_h_. The total field horizontal vector **T**_h_ will oscillate between **T***_h1_* and **T***_h2_* (where **T***_h_*_1_ = **T***_h_*_2_) and have nonzero azimuth α_1_, α_2_ (the angles between **B** and **T**_h_, α_2_ = 360° − α_1_). The resulting inclination angles β_1_ = β_2_ will be slightly different from the GMF inclination *I* at a distance of over 300 m from the wires. (**b**) In another idealized example, the dog stands under the wire strictly perpendicular to the magnetic NS axis. Both **W** and **T**_h_ are parallel to **B**_h_ (but **T***_h_*_1_ ≠ **T***_h_*_2_). The azimuth of **T**_h1_, **T**_h2_ is α_1_ = 0°, α_2_ = 180°. The inclination angles β_1_ ≠ β_2_ will significantly differ both from GMD inclination *I* and each other. (**c**) Schematic representation of the experiments in this study, where a dog stands under the wire, neither strictly parallel nor perpendicular to the NS axis (like power lines parallel to topographic, not magnetic, N-S axis). Here, **T***_h_*_1_ ≠ **T***_h_*_2_, α_1_ ≠ α_2_ and β_1_ ≠ β_2_ ≠ *I*.

The physical phenomena related to GMF and MF under high-voltage PL are depicted in Figure 1 and Figure 2. In the absence of artificial MF sources or strong natural anomalies, animals rely on GMF as a cue for orientation. GMF properties (direction, declination, inclination, and magnetic induction indicative of the field strength) are shown in Figure 1. High-voltage PL generates alternating MF at 50 Hz (the standard frequency in the Czech Republic). These fields reach their maximum intensities directly beneath PL, at mid-span between pylons, where conductor sag brings the lines closest to the ground: approximately 15 µT for 380 kV, 8 µT for 220 kV, and 5 µT for 110 kV lines [50,51]. At such locations, MF is the resultant total field **T**, defined as the vector sum of GMF (**B**) and the wire-generated MF (**W**) according to the superposition principle. **T** oscillates 50 times per second within a defined angular range determined by the relative orientation of PL relative to GMF (Figure 2). It seems possible that dogs can perceive this oscillating field, as the 50 Hz frequency falls within the neural processing range for both their visual and somatosensory stimuli [52]. However, it is more likely that the presence of multiple, rapidly changing magnetic signals deviating from magnetic north may interfere with their magnetic sense, causing alignment disruption through compromising either magnetically modulated visual patterns or the magnetic compass itself [16].

This study was designed to test the hypothesis that alternating MF generated by high-voltage PL disrupts spontaneous magnetic alignment in domestic dogs, as previously observed in cattle and deer [49]. To evaluate this, we recorded and analyzed the spontaneous body alignment of dogs during excretion (urination and defecation) under conditions with and without detectable ELF MF generated by overhead PL.

## 2. Materials and Methods

### 2.1. Data Collection

**Figure 3:**
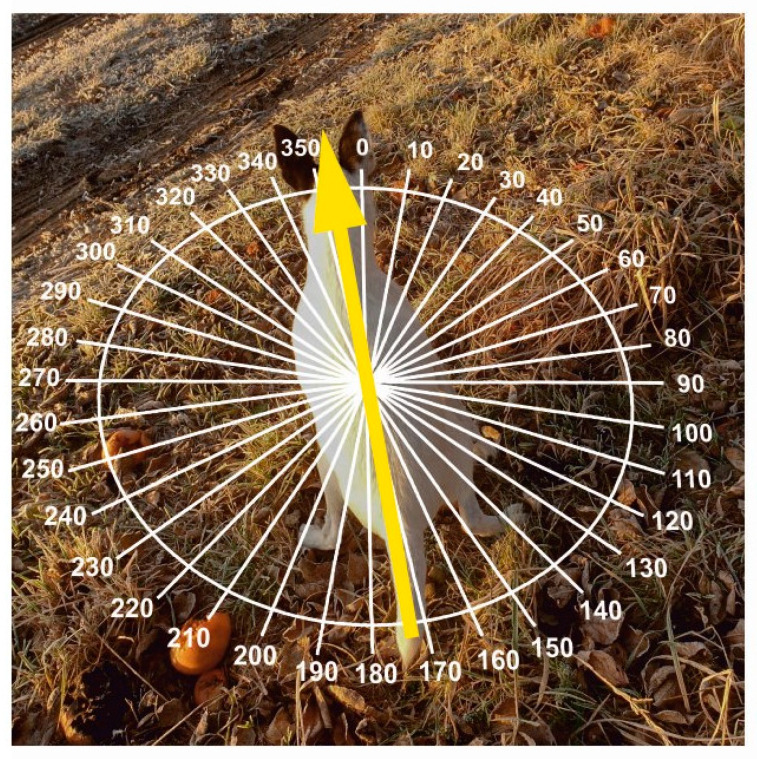
Body orientation of the dogs during the measurements. The bearings were recorded during the dog’s defecation or urination, measured as the azimuth of the axis going along the thoracic spine (between scapulae) towards the head. Photo credits: J. Adamková.

The study was conducted at 38 locations across the Czech Republic between 15 April 2013 and 25 June 2015 (Supplementary material, Table S1). We recorded the body alignment of 36 healthy dogs freely moving off the leash—23 males and 13 females belonging to 18 breeds and 3 crossbreeds, aged from 1 to 13 years (Supplementary Materials, Tables S2 and S6). Magnetic alignment was quantified as the angular position of the thoracic spine axis relative to magnetic north (Figure 3). Observations were made during the dog’s spontaneous urination and/or defecation, following the procedure described in [30]. Owners, trained in the protocol, recorded hand compass bearings (precision ±5°) while walking their dogs in designated areas. For each dog, 54–776 control measurements and 48–378 experimental measurements were collected throughout the day across the study period, yielding a total of 24,217 measurements. Data from [30] were excluded; all analyses are based solely on newly collected data.

In control conditions, dogs were roaming freely in open rural habitats (meadows, fields) located at least 500 m from potential confounding features such as linear structures or anthropogenic magnetic noise sources that could potentially affect dogs’ magnetic sense (such as fences, walls, motorways, powerlines, and large metal constructions). Experimental observations were conducted at comparable rural locations situated directly beneath high-voltage PL; dogs’ owners were instructed to perform the walks accordingly. Systematic measurements of alignment at distances of 10, 20, and 30 m from the power line wires (the approach used for cattle lying on the ground in [49] based on Google Earth screenshots) were not possible in this case. Bearings were taken when dogs were positioned under the PL wires at least 10 m away from pylons. Selected PL were oriented either north–south (NS) or east–west (EW) with respect to true (geographic, not magnetic) north, as determined using Google Maps. Creating sham PL (lines without current) was technically unfeasible for obtaining a statistically robust dataset.

For each measurement, the time (±1–10 min) and date of collection were recorded. Continuous magnetograms corresponding to all the observation dates were obtained from the Geomagnetic Observatory Fürstenfeldbruck in Munich, Germany http://www.geophysik.uni-muenchen.de/observatory/geomagnetism (last accessed on 1 December 2025) and used to calculate relative changes in GMF declination [30]. Time-specific GMF parameters (B*_x_*, B*_y_*, B*_z_*) for the experimental locations and dates were retrieved from the NOAA database https://www.ngdc.noaa.gov (last accessed on 1 December 2025). We used 20 µT and 50 Hz as standard values for power lines in the Czech Republic, as direct field measurements would be technically unfeasible and could distract the dogs.

### 2.2. Statistical Analyses

We conducted the procedures of first- and second-order circular statistics and generated graphs in Oriana v4.02 (Kovach Computing Services, 1994–2013). Additional statistical analyses were performed in R Studio v. 2023.03.0 (Posit Software, PBC, 2009–2023, packages *circular*, *CircStats*, *NPCirc* for the circular statistics, and *lme4* for mixed models of linearized angular data). MF vector calculations and visualizations were conducted in Mathematica 13.0 (Wolfram Research).

Previous work [30] demonstrated that the percentage of relative change in GMF declination (RCD) provides a more informative marker of geomagnetic conditions than the K*p* index for studies of spontaneous magnetic alignment. In this study, RCD was calculated as the difference between minimum and maximum of GMF declination during a defined disturbance period, expressed as a percentage of 360 arch minutes divided by the disturbance duration, as measured on magnetograms. For example, RCD = 2% means a change in declination equal to 1.2 arch min/h (1.2/60*100%) or 0.02°/h. RCD values were determined for each time point of data collection and were used to categorize angular measurements into ‘magnetic calm’ (RCD = 0%) or ‘magnetic disturbance’ (moderate, RCD ≤ 2%, strong, RCD > 2–3%).

Compass-based azimuth measurements differ from true north by a location-specific angle of declination (*D*). Consequently, the direction of PL wires in our observations did not perfectly align with, nor remain strictly perpendicular to, the GMF induction vector (**B**). Parameters of the total MF beneath PL were calculated using averaged values of B_x_, B*_y_*, and B*_z_* across the study period and across location groups (PL oriented geographically, rather than magnetically, NS or EW).

Given a compass measurement error of ±5°, raw angular data were binned into 10° classes. As an initial inspection, both the complete dataset and individual sub-datasets (each for one dog with > 10 measurements) were examined for evidence of bi- or multimodality; we applied visual observation and a likelihood ratio test for circular multimodality [53]. We calculated basic circular statistics (mean vector *µ*, mean vector length *r*, circular standard deviation *CSD*, and concentration parameter *k*), as well as second-order statistics–Grand Mean (GM) vectors, Moore’s Modified Rayleigh Test for non-uniformity of axial grouped data. One-sample test (Rayleigh’s Uniformity Test) was applied to assess departures from uniform distribution for individual dogs, while for GM, we used its Moore–Rayleigh modification as better performing for the data with measurement imprecision. As measures of scatter, we employed *r* as an indicator of concentration and *k* as a measure of dispersion [54,55].

Standard tests for comparing two circular means—Hoteling’s Two Sample Test and Moore’s Paired Test [56]—are not applicable to grouped circular data. To address this, we used a MANOVA-based approach recently proposed by [57]. This method linearizes circular values by converting each angle into sine and cosine components, which can then be analyzed using conventional MANOVA (R packages *stats*, *MANOVA.RM*) or linear models that accept paired data. For group comparisons, we applied a linear mixed-effects model to linearized circular data, designating individual dogs as a random factor, and including dog-specific characteristics (sex, age, weight, breed, and owner) as fixed factors.

## 3. Results

### 3.1. Differences in Alignment During Geomagnetic Calm and During the Periods of Geomagnetic Disturbances

**Figure 4:**
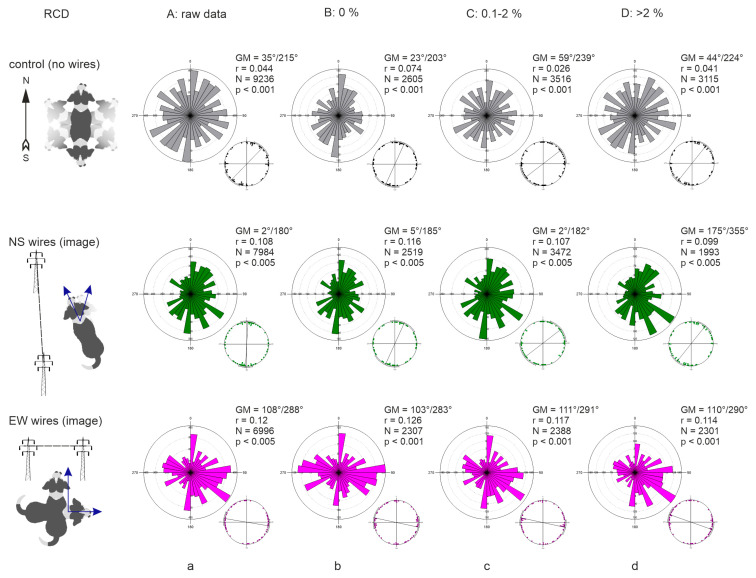
Directional alignment of freely roaming dogs in control and experiment. (**a**) Representation of all datapoints, regardless of the magnetic weather. (**b**) Alignment during the periods of magnetic calm (RCD = 0%). (**c**) Alignment during the periods of moderate magnetic disturbance (RCD ≤ 2%). (**d**) Alignment during strong magnetic disturbances (RCD > 2%). The NS arrow represents the direction of GMF. The line with pillars represents the direction of PL wires. Two blue arrows represent the direction of total field horizontal components **T***_h_*_1_, **T***_h_*_2_ under the wires. A rose diagram represents the distribution of all bearings for all dogs. The number on the top of each rose diagram represents the number of measurements within each directional bin. Smaller point diagram shows the distribution of means (one point—mean angle for one dog); the line represents the size and direction of Grand Mean (GM) vector with 95% CI *r*; GM—axial Grand Mean angles; N—total number of measurements for each condition; *r*—length of the mean resultant vector of individual directional vectors; *p*—GM significance according to Moore’s Modified Rayleigh Test with weighted means.

The distribution of bearings in both control conditions and beneath PL wires was axial as suggested by visual inspection and multimodality test (Figure 4). The Grand Means (GM) calculated from the individual axial means (each mean is calculated from >50 bearings of one dog) at any RCD were 35°/215° in control (36 dogs), 2°/182° under NS-directed PL (36 dogs), and 108°/288° under EW-directed PL (34 dogs). Overall, bearings were widely scattered around 360° (Figure 4a). However, during the periods of magnetic calm (RCD = 0%), bearings became more concentrated along the NS or EW axis (Figure 4b): GM was 23°/203° in control, shifting to 5°/185° under NS-oriented PL, and to 103°/283° under EW-oriented PL. Interestingly, the distribution of mean angles under EW-directed PL was trimodal (Likelihood ratio test for multimodality: estimated number of nodes = 3, *p* = 0.042).

In control, GM deviated progressively from the NS axis (relative to magnetic N) toward the EW axis with increasing geomagnetic disturbances, when RCD changed from 0% to 2% and higher (Table 1, Figure 4b–d). These directional changes were highly significant (LME: F = 6.12, *p* = 0.0022). In contrast, under NS-oriented PL wires, GM changed only slightly (Table 1), with differences that were not significant (F = 0.23, *p* = 0.79). Under EW-oriented PL, GM showed a minor clockwise shift, but again, changes were not significant (F = 0.59, *p* = 0.55).

To evaluate the effect of geomagnetic disturbances on the concentration of bearings, we used standard circular measures: mean vector length (*r*) and concentration parameter (*k*). Under control conditions, both *r* and *k* decreased with increasing geomagnetic disturbances (Table 1), indicating greater dispersion of bearings during disturbed periods. These reductions were statistically significant (LME: F_r_ = 4.749, *p* = 0.012; F*_k_*: = 5.348, *p* = 0.007). Under NS-oriented PL, *r* and *k* showed only minor, non-significant changes (LME: F*_r_* = 0.852, *p* = 0.43; F*_k_*: = 1.885, *p* = 0.16). Under EW-directed PL, *r* and *k* decreased more markedly (*k* = 0.322), but these changes were likewise non-significant (LME: F*_r_* = 0.573, *p* = 0.57; F*_k_*: = 0.573, *p* = 0.99).

### 3.2. Predictions for the Total Magnetic Field Direction Under the Power Lines

As described above, beneath PL wires, the horizontal component of the total field (**T**_h_) is the vector sum of the GMF horizontal component (**B***_h_*) and the field generated by the wire (**W**, ~20 µT in the studied power lines) that is lacking vertical component. **T**_h_ alternates symmetrically around its median at a frequency ~50 Hz. Across all observation sites, the total GMF ranged from 44.1792 to 49.1076 µT, with field parameters summarized in Table 2 (see Supplementary material, Table S2 for location details). Mean values of magnetic induction, declination (*D*, α_0_), and inclination (*I*, β_0_) were calculated for each condition (Table 2).

In the control locations (no PL):

**B**_ctr_ = (B_x_, B_y_, B_z_) = (19.9830, −1.2373, 44.5499), |B_ctr_| = 48.8982 µT, *D*_ctr_ = 3.55°, *I*_ctr_ = 65.81°.

For locations with NS-oriented PL (outside the impact of PL):

**B**_NS_ = (B_x_, B_y_, B_z_) = (19.9596, −1.2149, 44.6225), |B_NS_| = 48.8157 µT, *D*_NS_ = 3.48°, *I*_NS_ = 65.86°.

For locations with EW-oriented PL (outside the impact of PL):

**B**_EW_ = (B_x_, B_y_, B_z_) = (19.9784, −1.1925, 44.6184), |B_EW_| = 48.9015 µT, *D*_EW_ = 3.41°, *I*_EW_ = 65.84°.

**Figure 5:**
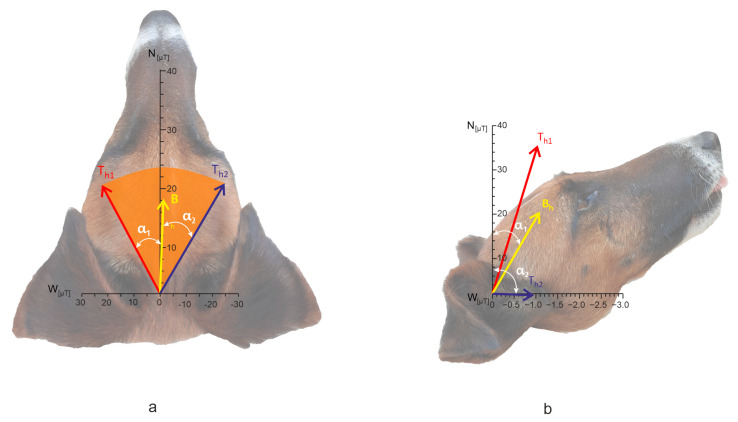
Horizontal component of the total field **T** under the high-voltage PL versus dog’s alignment (2D representation of 3D system). (**a**) total field under NS-directed PL; (**b**) total field under EW-directed PL (the angles are scaled according to the 3D positions of the vectors). N, W—magnitudes of the corresponding components, figures on the scale indicate magnetic induction in µT. **B***_h_*—horizontal component of GMF at the given location away from the wires, **T***_h1_*, **T***_h2_*—alternating horizontal component of the total field, designation of α_1_, α_2_ correspond to that in Figure 2. The dog’s head in the background is included for illustrative purposes only.

We then calculated the magnetic induction of the total field **T** = **B** + **W** separately for NS- and EW-oriented PL (Table 2, Figure 5). Directly beneath NS-oriented PL, the horizontal component of **T** alternated between **T***_h_*_1_ = 27.4092 µT and **T***_h_*_2_ = 29.1283 µT, with the amplitude angle Dα = 45.0° (Figure 5a, the vector lengths and the angles are scaled). The median azimuth between α_1_ and α_2_ was 356.5°. Inclination angles (not shown in Figure 5 but listed in Table 2) deviated from *I*_NS_ by −32.7° and −34.3°, yielding Dβ = 1.6°. The resulting inclination of **T** was shallower than that of GMF (*I*).

Directly beneath EW-oriented PL, the horizontal component of **T** alternated between **T**_*h*1_ = 39.9961 µT and **T**_*h*2_ = 1.1907 µT, Dα = 89.33° (Figure 5b). The mean angle between α_1_ and α_2_ was 43.8°. The left extreme of **T** was oriented approximately north and the right extreme toward the east. Inclination angles β_1_ and β_2_ deviated from *I*_EW_ by −17.7° and 21.1°, respectively, with Dβ = 38.8°.

### 3.3. Impact of Power Lines on Dogs’ Alignment

To test the hypothesis that alignment would be disrupted (and the bearings scattered) beneath PL, we used *r* and *k* as the measures of concentration/dispersion of bearings, and compared these two parameters calculated from control data with those characterizing the alignment under the PL.

As shown in the previous section, total *r* and *k* values calculated from bearings recorded under PL were higher than those in the control. This indicates that dogs’ alignment did not disappear under PL; to the contrary, the bearings were less scattered: total *r*_control_ = 0.064, *r*_NS_ = 0.125, *r*_EW_ = 0.124; total *k*_control_ = 0.127, *k*_NS_ = 0.251, *k*_EW_ = 0.251. These differences were statistically significant (LME: F*_r_* = 22.328, *p* = 0.0017; F*_k_* = 19.471, *p* = 0.0024).

Nevertheless, marked changes in the alignment pattern and direction were observed beneath high-voltage PL. In control conditions, dogs oriented primarily along 23°/203° (relative to magnetic north) during geomagnetic calm, with bearings concentrated along the NS axis relative to magnetic N (Figure 4b; Supplementary Materials, Tables S3–S5). Under geographically NS-oriented PL, the bearings remained concentrated along the magnetic NS axis (GM = 5°/185° relative to magnetic N, RCD = 0%). In contrast, under geographically EW-oriented PL, dogs aligned either along the NS axis or perpendicular to it (trimodal distribution, GM = 103°/283° relative to magnetic N, RCD = 0%). This difference in alignment was highly significant (Linear Mixed-Effects Model for linearized angles: F = 48.758, *p* < 0.001 for control vs. NS PL, F = 90, *p* < 0.001 for control vs. EW PL).

## 4. Discussion

Research on magnetoreception in large mammals remains challenging, as controlled laboratory experiments involving animal immobilization or direct brain electrophysiological recordings are ethically and technically unfeasible. Experiments with dogs in large magnetic coils have produced promising results [58], although these methods require further refinement. As an alternative, broad-scale observations of animals in natural settings provide a viable approach, particularly when individuals display spontaneous behaviors previously demonstrated to reflect magnetic orientation [30,42,45]. In this study, we analyzed a large dataset (>24,000 measurements) collected under conditions where magnetically mediated behavior was expected to be either unaffected (control) or influenced by potentially disruptive factors, specifically the alternating MF generated by high-voltage PL.

The phenomenon of dogs’ spontaneous alignment along the NS axis under calm geomagnetic conditions was first described in [30], in observed dogs “moving freely,” i.e., not on a leash, in open-field environments (meadows, fields, and occasionally woodland) in the absence of prominent directional cues (roads, paths, PL) or major distractions (strangers or other dogs). Later, two independent studies conducted in urban dog park areas [33,59] demonstrated that experiments performed in densely populated environments and by personnel lacking sufficient training may yield contradictory results. To avoid such confounding effects, we conducted a blind study with trained adult observers. Control observations were made in rural open areas (meadows and fields) free of directional cues (including small paths), unfamiliar people, and other dogs. Comparison of dogs’ body alignment across control conditions and under PL oriented along NS or EW axes showed that high-voltage PL induced directional changes but did not disrupt alignment, in contrast to the disruption reported in cattle and deer [49]. Some scattering of bearings was observed when dogs were exposed to PL during geomagnetic disturbances; however, this scattering was much less pronounced than under control conditions. The most straightforward explanation of this phenomenon would be that, in the absence of a static, non-oscillating magnetic cue of the GMF intensity, dogs may switch completely from magnetoreception to relying on prominent visual cues for orientation, and align accordingly (for example, in the direction of PL pylons). However, this explanation does not account for the trimodal alignment pattern observed under EW-oriented PL that contains NS and EW components (Figure 4b, EW wires), which is different from orienting observed under EW-directed PL in the domestic cattle.

The geomagnetic environment at the time of recording can significantly influence animal behavior and physiology [60,61], including directional alignment in dogs [30]. Under geomagnetic calm and in the absence of non-geomagnetic MF sources, dogs exhibit spontaneous alignment along the NS axis. As it was demonstrated both in [30] and in this study, during magnetic disturbance, such alignment significantly departs from the NS axis, introducing additional information noise that can be a source of false positives and false negatives. Since there is variation in direction and strength of GMF, including small reversible variations due to solar activity, only the days and times of magnetic calm should be selected to evaluate the significance of other factors in directional alignment.

The sensitivity of dogs to even minor GMF disturbances, such as rapid GMF declination changes of ≥0.02°/h [30], suggests an ability to detect weak MF and their subtle directional changes. Prior to this study, it remained unclear whether dogs can perceive alternating MF or discriminate between two oscillating MF vectors. Our results confirm the conclusions of [30] that in the absence of PL and during geomagnetic calm, dogs aligned roughly along the NS axis (Figure 4b, control/no wires; GM = 23°/203°, Moore–Rayleigh Z = 1.976, *p* < 0.001), but this alignment changed during geomagnetic disturbances (Figure 4d control/no wires; GM = 44°/224°, Moore–Rayleigh Z = 1.933, *p* < 0.001). Under NS-oriented PL, dogs predominantly aligned in NS direction during magnetic calm periods (Figure 4b, NS wires; GM = 5°/185°, Moore–Rayleigh Z = 1.446, *p* < 0.005), consistent with the total field median direction (median of the two alternating MF vectors beneath the wire) and PL orientation. In contrast, under EW-oriented PL two perpendicular alignments were observed during geomagnetic calm conditions (Figure 4b, EW wires) roughly correspond to the two alternating MF vectors beneath the wire, and PL orientation (GM = 103°/283°, Moore–Rayleigh Z = 2.263, *p* < 0.001). In [30], dogs’ alignment during defecation (mean μ = 173°/353°) and urination (μ = 167°/347°) was slightly shifted toward the north–northwest during the periods of geomagnetic calm. In our study, we analyzed defecation and urination data jointly, assuming a shared underlying orientation mechanism. Under geomagnetic calm, dogs in our control group showed a summarized alignment with a slight north–northeast shift. This difference may reflect effects of breed, training, and age, as recently discovered by [62], since our study used a different cohort of animals than [30].

The observed patterns seem to be more complex than a simple shift from magnetoreception to reliance on visual cues. (e.g., pylons as the overhead wires are not clearly visible for roaming dogs and do not constitute a simple directional cue). We excluded scent marks from other animals (domestic dogs, cattle, or wild species) as potential directional cues, as their distribution within the study areas would be random rather than systematically aligned with cardinal directions. According to observer reports, there were also no prominent sounds detected that could have been interpreted as directional indicators. Another, although less parsimonious explanation may be that dogs combine both visual and magnetic (even if oscillating) cues for orientation. As highlighted in the review on the ‘noisy’ nature of animal magnetoreception [63], non-magnetic cues may complement the magnetic sense. Experimental evidence from Bogong moths [64] supports such a multimodal orientation system, in which magnetic input is integrated with visual cues. Similarly, avian navigation is as well established as multimodal, relying on sun, stars, and magnetic compass [20,65].

The temporal resolution of the cone cells in the canine retina is higher than in humans, reaching 70–80 Hz, while the critical flicker fusion of rod cells is similar in both species (~20 Hz) [52]. This suggests that dogs may be able to detect, albeit during daylight, visual flickering at frequencies above 50 Hz. However, it would be speculative to assume that they can also perceive MF oscillating at this frequency as a discrete cue, or use it for orientation, and further research is needed on this matter.

The intensity of the total field **T** (Figure 5, Table 2) in this study was about 1.2 times higher than that of B, exceeding by far the sensitivity range of the inclination compass estimated for birds [66]. Unfortunately, because we did not record the dogs’ alignment separately on the left and right sides of the wires, as described for cattle in [49], this study does not allow us to determine whether dogs employ a radical-pair–based inclination compass, neither if the observed increase in field intensity (**B** + 76%) could render the dogs’ magnetic compass non-functional, thereby narrowing the potential causes of the persisting alignment to visual or other cues (e.g., position of the Sun). Further research is needed to clarify the underlying reasons for the persistence of dogs’ alignment under PL.

## 5. Conclusions

1. Our study provides further evidence of magnetoreceptive abilities in domestic dogs, expressed, among other behaviors, as spontaneous directional alignment. This alignment occurs consistently in the absence of magnetic sources other than the geomagnetic field (GMF) and demonstrates dogs’ sensitivity to minor variations in GMF declination.
2. Under the PL wires, dogs were exposed to an oscillating magnetic stimulus 1.24 times more intensive than the GMF, alternating at 50 Hz, in a direction differing from the GMF vector (41.5° and 311.5° relative to magnetic north under NS-directed PL, and 91.0° and 358.3° under EW-directed PL).
3. Dogs maintain directional alignment under PL, unlike cattle and deer [49]. While the simplest explanation of this phenomenon would be a shift from magnetic to visual cues (like PL pylons) for orientation, the trimodal pattern observed under EW-directed PL (with both NS and EW components) suggests a more complex mechanism, not excluding a combination of magnetic and non-magnetic cues.

## Figures and Tables

**Table 1:** Characteristics of dogs’ alignment during magnetic calm and geomagnetic disturbances. Axial GM—Grand Mean of alignment angles calculated for axial data; *r*—length of the mean direction vector; *k*—estimate of bearings concentration.

Direction of the Wires	Magnetic Calm (RCD = 0%)			Moderate Magnetic Disturbances (RCD = 0.1–2%)			Strong Magnetic Disturbances (RCD > 2%)		
	Axial GM	*r*	*k*	Axial GM	*r*	*k*	Axial GM	*r*	*k*
No wires	23°/203°	0.074	0.214	59°/239°	0.026	0.086	44°/224°	0.041	0.119
Geographic NS	5°/185°	0.149	0.301	5°/185°	0.113	0.228	2°/182°	0.119	0.241
Geographic EW	103°/283°	0.159	0.322	111°/291°	0.101	0.203	110°/290°	0.137	0.277

**Table 2:** Magnetic field characteristics in the control (no high-voltage PL) and the experiments (NS- or EW-going PL). N—number of localities.

Direction of the Wires	N	$\mathrm{Parameters}\;\mathrm{of}\;\mathrm{GMF}\;(B)\;(\bar{x}\pm \mathrm{SD})$			Total Field (*T*) Characteristics					
		B_x,_ µT	B_y,_ µT	B_z,_ µT	|*T*_1|,µT_	|*T*_2|, µT_	α_1_	α_2_	β_1_	β_2_
No wires	26	19.98 ± 0.15	1.23 ± 0.14	44.62 ± 0.18	|B| = 48.9 µT, B_h_ = 20.0 µT, *D*(α_0_) = 3.6°, *I* (β_0_) = 65.8°					
Geographic NS	15	19.96 ± 0.13	1.23 ± 0.11	44.64 ± 0.15	52.4	53.3	41.5°	311.5°	33.12°	31.6°
Geographic EW	14	19.97 ± 0.14	1.19 ± 0.12	44.60 ± 0.17	59.9	44.7	91.0°	358.3°	48.1°	87.0°

## Data Availability

The original data analyzed in this paper are publicly available on Dryad repository and can be downloaded at: https://datadryad.org/share/e2T2311ESuJoahA8mdyrwBoWoE8257ty5NIQMNOvFs0 (accessed on 1 December 2025).

## Supplementary Materials

The following supporting information can be downloaded at: https://datadryad.org/share/e2T2311ESuJoahA8mdyrwBoWoE8257ty5NIQMNOvFs0 (last accessed on 1 December 2025), Tables S1–S5. Information about the dogs used in the experiments, locations, and basic statistics; Table S6. Raw measurements of dogs’ alignment in control and under PL.

## Abbreviations

The following abbreviations are used in this manuscript:

GMF	Magnetic field of Earth/geomagnetic field
MF	Magnetic field
PL	Power lines
NS	North–south (direction)
EW	East–west (direction)
